# Entanglement of UPR^ER^ in Aging Driven Neurodegenerative Diseases

**DOI:** 10.3389/fnagi.2017.00341

**Published:** 2017-10-24

**Authors:** Safikur Rahman, Arif Tasleem Jan, Archana Ayyagari, Jiwoo Kim, Jihoe Kim, Rinki Minakshi

**Affiliations:** ^1^Department of Medical Biotechnology, Yeungnam University, Gyeongsan, South Korea; ^2^Department of Microbiology, Swami Shraddhanand College, University of Delhi, New Delhi, India; ^3^Institute of Home Economics, University of Delhi, New Delhi, India

**Keywords:** aging, UPR (unfolded protein response), endoplasmic reticulum (ER), neurodegenerative diseases, dementia

## Abstract

The endoplasmic reticulum (ER) is an indispensable cellular organelle that remains highly active in neuronal cells. The ER bears the load of maintaining protein homeostasis in the cellular network by managing the folding of incoming nascent peptides; however, the stress imposed by physiological/environmental factors can cause ER dysfunctions that lead to the activation of ER unfolded protein response (UPR^ER^). Aging leads to deterioration of several cellular pathways and therefore weakening of the UPR^ER^. The decline in functioning of the UPR^ER^ during aging results in accumulation of misfolded proteins that becomes intracellular inclusions in neuronal cells, resulting in toxicity manifested as neurodegenerative diseases. With ascension in cases of neurodegenerative diseases, understanding the enigma behind aging driven UPR^ER^ dysfunction may lead to possible treatments.

## Introduction

The cellular homeostasis maintains existence of life through integrative communication among various macromolecules working in unity through numerous biochemical pathways. The endoplasmic reticulum (ER) not only maintains Ca^2+^ homeostasis but also controls translation, folding, maturation and trafficking of about one third of cellular proteins. Various environmental insults can disturb proper functioning of ER, leading to accumulation of unfolded/misfolded protein cargo in the ER lumen that gives rise to a condition called ER stress. The cell responds through a highly conserved pathway known as the ER unfolded protein response (UPR^ER^; Walter and Ron, 2011; Corazzari et al., 2017). UPR^ER^ first focuses on alleviation of the imposed stress by initiating steps of adaptive mechanisms in the secretory pathway for restoration of homeostasis but conditions of prolonged stress and damage provokes UPR^ER^ to succumb through apoptosis (Walter and Ron, 2011).

Aging is notably a process during which the cell witnesses decline in its ability to respond to stress. Age related frailty perturbs the multifarious schematic of UPR^ER^ giving rise to a myriad of pathologies characterized by the presence of disease specific misfolded proteins playing havoc with cellular homeostasis (Nuss et al., 2008). The pathology of neurodegenerative disorders such as Alzheimer’s disease (AD), Parkinson’s disease (PD) and Huntington’s disease (HD) emerge as a consequence of disturbance in proteostasis. This review presents research findings that highlight the mechanism of UPR^ER^ in aging driven neurodegenerative diseases.

## The Pathway of ER Stress Induced UPR^ER^

**Figure 1:**
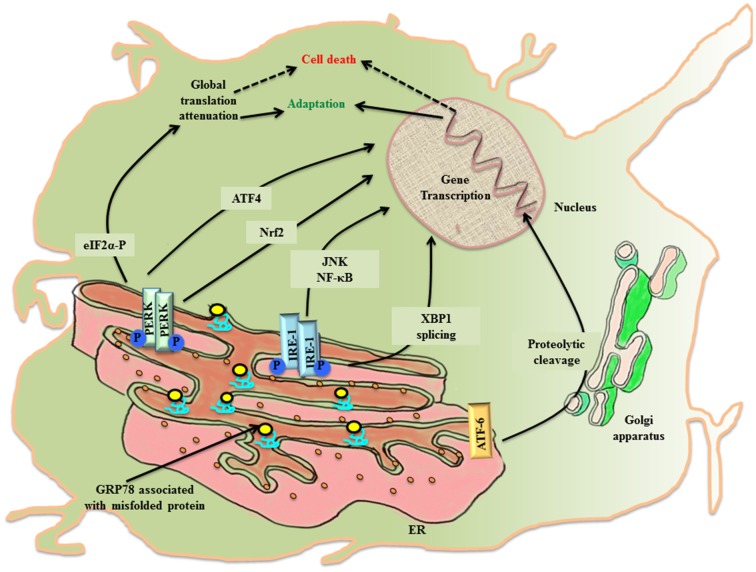
The activation of UPR^ER^ in the neuronal cell. Under the imposed ER stress, a neuronal cell activates UPR^ER^ that starts with the release of GRP78 from its association with the luminal components of the three transmembrane transducers of UPR^ER^: PERK, IRE1 and ATF6. GRP78 is recruited to the misfolded protein cargo. This stimulates all the three transducers in a series of events that disseminate their effect through transcriptional control of genes. PERK phosphorylates cytoplasmic eIF2 α that causes attenuation of global protein translation, paradoxically favors translation of ATF4 and activates Nrf2. Further IRE1 leads to XBP1 splicing and activation of JNK/NF-κB. ATF6 undergo proteolysis. All working in coherence, first ameliorates the stress, but later under chronic ER stress apoptotic pathway leads to cell death.

The ER lumen docks a range of resident molecular chaperones like glucose regulated protein 78 (GRP78), glucose regulated protein 94 (GRP94), caltericulin (CRT) and protein disulfide isomerase (PDI) that aid in folding of incoming nascent proteins. GRP78, also referred to as BiP/HSPA5, is the master ER chaperone that folds the nascent polypeptides, binds the ER luminal Ca^2+^ and marks misfolded protein cargo for their degradation through ER associated degradation (ERAD; Lee, 2005; Wang et al., 2009; Park et al., 2017). During ER stress, ER lumen is overloaded with misfolded protein, so the molecular programming of UPR^ER^ first tries to alleviate the debilitated homeostasis by upregulating the expression of GRP78 (Zhu and Lee, 2015). Normally, GRP78 rests in association with the luminal components of three ER resident transmembrane proteins referred to as the sensors of UPR^ER^: PKR-like ER kinase (PERK), inositol requiring enzyme-1 (IRE-1) and activating transcription factor 6 (ATF-6). First, an adaptive response of UPR^ER^ starts where GRP78 disassociates from the luminal components of transmembrane PERK, IRE-1, ATF6 and gets recruited to the misfolded protein cargo. This activates UPR^ER^ signaling pathway that disseminates the information along a cascade of downstream effector molecules in the cytosol (Figure 1).

PERK undergoes homo-dimerization and trans-autophosphorylation (PERK-P) upon leaving its association from GRP78. The activated PERK phosphorylates on serine 51 of the cytoplasmic eukaryotic initiation factor 2α (eIF2 α; eIF2 α-P) which inhibits its immediate effector, guanine nucleotide exchange factor for eIF2 complex, thereby preventing the assembly of 43S initiation complex. This leads to attenuation of global protein translation in the cell aiming to reduce the load of new protein cargo in the ER lumen, but paradoxically favors the translation of mRNA with internal ribosome binding sites (IRES) such as activating transcription factor 4 (ATF4; Harding et al., 2000). ATF4 is a cAMP response element binding (CREB) transcription factor that is involved in controlled up-regulation of genes for amino acid metabolism, antioxidant response, autophagy and apoptosis (Ma and Hendershot, 2003; Blais et al., 2004). The ATF4 mRNA has two upstream open reading frames (uORFs), uORF1 and uORF2, in 5′ untranslated region (UTR), whose translation depends upon the concentration of eIF2-GTP- Met-tRNA_i_^Met^/40S ribosome ternary complex. In non-stressed condition, low levels of eIF2 α-P and ample ternary complex concentration, leads to the translation of both uORFs and attenuation of coding ATF4 transcript (Vattem and Wek, 2004). Whereas stress induced rise in concentration of eIF2 α-P diminishes ternary complex prompting translation of coding ATF4 transcript (Baird et al., 2014). Under the condition of unresolved ER stress, ATF4 expression is prolonged, which ultimately up-regulates another effector molecule, C/EBP homologous protein (CHOP), thereby stimulating the apoptotic-signaling cascade. PERK arm of UPR^ER^ affects antioxidant pathway, not only through ATF4 activation, but also via another cytoplasmic substrate, the nuclear factor E2 related factor2 (Nrf2), which gets imported into the nucleus for up-regulation of antioxidant genes (Cullinan et al., 2003).

The stimulation of IRE1 after its dissociation from GRP78 during the imposed ER stress leads to homo-dimerization and autophosphorylation of IRE1 (IRE-P). This imposes conformational change in IRE1 that affects its downstream effector molecules. To release the stress imposed on ER lumen due to overload of protein folding, the endoribonuclease activity of phosphorylated IRE1 splices X-box binding protein-1 (XBP1) in an unconventional way producing XBP1(S), which is ensued by the activation of genes for UPR^ER^ like chaperones, ERAD and organelle biosynthesis (Yoshida, 2007). In another attempt to rescue the cell, IRE1 independently degrades specific subsets of mRNA, thereby halting production of new proteins in the ER lumen (Hollien and Weissman, 2006). However, when the imposed stress continues to impede the system, the activated IRE1 arm also participates in triggering pro-apoptotic signaling by forming complex with TNF receptor-associated factor 2 (TRAF2), leading to activation of c-jun-N-terminal kinase (JNK; Urano et al., 2000; Nishitoh et al., 2002).

Upon activation, the third arm of UPR^ER^, ATF6, is translocated to the golgi apparatus, where it undergoes intramembranous proteolytic cleavage by site-1 and site-2 proteases (S1P and S2P respectively). This event releases N-terminal part of ATF6 that is imported into the nucleus to upregulate the transcription of ER stress responsive elements (ERSE; Lee et al., 2002).

The eIF2 α-P also leads to the activation of another closely related stress response referred to as the integrated stress response (ISR). The PERK arm of UPR^ER^ joins hands with ISR through eIF2 α-P, which is also the target of three other kinases of the pathway: RNA-activated protein kinase (PKR), heme-regulated inhibitor kinase (HRI) and general control non-depressible 2 (GCN2). The attenuation of most of the cellular mRNA translation after phosphorylation of eIF2 α saves the cell by conserving molecular resources in the cytosol (Palam et al., 2015).

## UPR^ER^ Intersects with Inflammation and Autophagy

The master transcriptional regulator of inflammation nuclear factor-κB (NF-κB) has been reported to be upregulated during ER stress (Deniaud et al., 2008). The tripartite arm of UPR^ER^ touches inflammatory signaling cascade directly/indirectly through NF-κB. The PERK/eIF2 α-P induced attenuation of global protein translation triggers NF-κB (57). The IRE1-TRAF2 complex not only activates NF-κB (Hu et al., 2006), but also JNK, during ER stress. In a study of Shiga toxigenic *E. coli*, ATF6 has been shown to cause ascension in the expression levels of NF-κB in reply to UPR^ER^ (Yamazaki et al., 2009).

Autophagy (macro-autophagy), in general, is a conserved survival pathway that canonically triggers degradation of organelles/molecules aimed at retrieving their building molecules back in the cytosol. During ER stress, UPR^ER^ effector molecules crosstalk with molecular markers of autophagy, the microtubule associated protein1 light chain 3 (LC3) and lysosome associated membrane protein (LAMP; Tanida et al., 2008). The PERK/eIF2 α-P/ATF4 and IRE1/spliced XBP1 pathways stimulate induction of LAMP-3 and LC3, respectively (Mujcic et al., 2009; Margariti et al., 2013). The induction of autophagy during UPR^ER^ potentiates the adaptive trial of the cell by clearing off the load of misfolded protein from ER lumen.

## Aging Abates Activation of UPR^ER^ and its Effectors

The process of aging causes decline in the proper functioning of cellular metabolic pathways. The changes in cells undergoing aging weaken UPR^ER^, causing it to fail to recuperate ER stress. The various molecular chaperones in the ER lumen, such as GRP 78, GRP 94, calreticulin and PDI, undergo oxidative damage in the aging cell that diminishes the efficiency of these molecular chaperones to fold nascent protein; hence, presenting a mass of misfolded protein cargo in the lumen (Rabek et al., 2003; Nuss et al., 2008). As evidenced through studies, the expression levels of GRP78 also become mitigated because of aging in murine cortex, rat hippocampus, cortex and cerebellum (Paz Gavilán et al., 2006; Hussain and Ramaiah, 2007; Naidoo et al., 2008). This causes protein toxicity, leading to derangement in proteostasis, which becomes an underlying cause of age related diseases.

Aging deteriorates the three molecular sensors of UPR^ER^. RT-PCR studies in aged mice showed significant lowering of PERK mRNA expression in rat hippocampus (Paz Gavilán et al., 2006). During aging cell environment starts favoring apoptotic-signaling cascade via activation of CHOP and caspases-12 (Hussain and Ramaiah, 2007; Naidoo et al., 2008). Furthermore, the IRE1 arm favors upregulation of kinases involved in apoptotic pathway, such as ASK1 and JNK (Ichijo et al., 1997). The molecular pathway of UPR^ER^ also intersects with inflammatory pathways in the cell (Cao et al., 2016). The master molecule NF-κB, is shown to be upregulated during aging (Yalamanchili et al., 2016). Aging associated human pathology has also been shown to involve a decline in autophagy (Caramés et al., 2010). Thus, aging imposes misfolded protein toxicity in the cell through disabled UPR^ER^, leading to emergence of age related dysfunction and diseases.

## The Molecular Signatures of UPR^ER^ in Aging Driven Neurodegenerative Diseases

**Figure 2:**
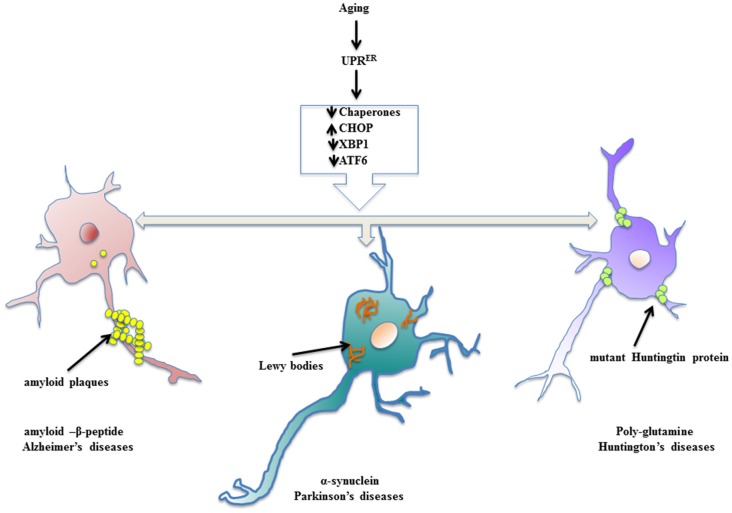
Enervated UPR^ER^ resulting in neuropathologies. Aging declines the function of UPR^ER^ thereby preparing the stage for surfacing of neurodegenerative diseases. The specific toxic, misfolded protein accumulations are characteristic of aging driven neurodegenerative diseases.

Neurodegenerative diseases find their source of origin in the perturbations that alter proper functioning of ER. Age related frailty disarms the adaptive arm of UPR^ER^ and presents distressing conditions in the brain to promote accumulation of misfolded protein cargo in the ER lumen that later on become inclusions of specific abnormal proteins. Most of the models of aging driven neurodegenerative disease have been marked with the presence of specific protein inclusions because of ER stress in the brain and central nervous system, which are toxic to the post-mitotic neurons. The evoked UPR^ER^ attempts to ameliorate the condition of deranged protein homeostasis, but failing to do so it compromises with the system in the form of neuropathology (Figure 2). During such proceedings, the synaptic loss becomes an early event in the pathology that conclusively leads to the death of neurons, which has been studied in cases of AD and PD and frontotemporal dementia (Mallucci et al., 2007; Tampellini, 2015). Here, we discuss neurodegenerative diseases with aging as the prominent risk factor in which the involvement of UPR^ER^ markers has been well studied and shown to be potential targets for therapeutic interventions.

## Alzheimer’s Disease (AD)

The most prominently studied form of dementia in aging patients is AD, which shows a characteristic extracellular buildup of toxic amyloid-β peptide (Aβ), hyperphosphorylated tau protein, which interfere with Ca^2+^ homeostasis and proteostasis, leading to synaptic loss and neuronal degeneration (Singh et al., 2016). Hoozemans et al. (2009) have shown in autopsy samples of brains of AD patients that ER stress markers like PERK-P, eIF2 α-P and IRE1-P markedly increased as a result of UPR^ER^ activation. Studies in AD temporal cortex and hippocampus registered two-fold heightened-expression levels of GRP78 (Milisav et al., 2015; Casas, 2017). Also in mice AD model, 1.5–2 fold increase in GRP78 was found to be associated with accumulation of Aβ (Soejima et al., 2013). Resende et al. (2008) studied the activation of UPR^ER^ in primary rat embryo cortical neurons treated with Aβ oligomers and documented significant increase in levels of GRP78 as well as ER Ca^2+^ release resulting in tau phosphorylation. Moreover, studies on persistent UPR^ER^ have shown the active involvement of glycogen synthase kinase 3β (GSK-3β), an active kinase involved in tau phosphorylation (Kim et al., 2005), which also co-localizes with PERK-P in cortical cells (Hoozemans et al., 2009), is a well-documented target of PERK (McAlpine and Werstuck, 2014). Additionally, tau protein has been shown to be involved in the stimulation of PERK, IRE1 and ATF6, which cooperatively elicit the inflammatory signaling cascade in the brains of AD patients (McAlpine and Werstuck, 2014) The chemical modifications of already prevailing proteins regulate short-term memory, whereas long-term memory requires *de novo* protein synthesis and is under the control of phosphorylation status of eIF2 α (Kandel, 2001). Cases of AD patients have shown increasing levels of eIF2 α-P in their histological samples (Hoozemans et al., 2007).

Studies on knock-in mice expressing mutant presenilin 1 (PS1), which induces early onset of familial AD, showed intensified levels of pro-apoptotic CHOP with concomitant diminishing levels of anti-apoptotic Bcl-2 (Milhavet et al., 2002). The role of active NF-κB in driving inflammatory gene transcription has been well documented in aging cell lines, as well as neuropathology in AD (Lukiw and Bazan, 1998).

Moreover, higher levels of LC3 have been reported in the hippocampal neurons in the brain of AD patients, suggesting the active involvement of autophagy (Nijholt et al., 2011).

## Parkinson’s Disease (PD)

The pathology behind PD has been speculated to be due to mutations in three different genes and certain specific transposons; namely, α-synuclein, associated with early onset of familial PD; parkin and ubiquitin C-terminal hydrolase L1, manifesting some rare forms of PD (Shen et al., 2016). The loss of dopaminergic (DA) neurons account for the motion disorder featured in PD (Hirsch et al., 2013). Lewy bodies (LBs), the heavily ubiquitinated cytoplasmic accumulations of α-synuclein, hallmark of PD and accumulations of parkin substrate due to loss of functional Parkin, cause ER stress that evokes UPR^ER^ (Imai et al., 2001). The increasing neuronal death in neurotoxin induced models of Parkinsonism has been reported due to apoptotic pathway, where robust expression of CHOP has been well documented (Silva et al., 2005), demonstrating the involvement of PERK arm of UPR^ER^. Paradoxically, the other two arms of UPR^ER^, IRE1 and ATF6, have been shown to be pro-adaptive and neuroprotective for DA neurons in cases of neurotoxin induced PD models through up-regulation of expression of GRP78 and ERAD genes (Egawa et al., 2011; Hashida et al., 2012; Valdés et al., 2014). In *Drosophila* model of PD expressing human α-synuclein, the protective arm of UPR^ER^ invariably coordinates through XBP1 mediated autophagy (Fouillet et al., 2012).

## Huntington’s Disease (HD)

HD is characterized by neuronal dysfunction and neurodegeneration in the CNS that leads to dementia. Brain samples from Huntington’s patients display accumulation of misfolded mutant Huntingtin protein (mHtt) as intracellular inclusions, where the glutamine residue shows an expansion of more than 40 repeats (Bossy-Wetzel et al., 2008). Reports suggest that UPR^ER^ is stimulated in HD cases. For example, increasing levels of GRP78 and CHOP mRNA have been observed in human autopsy samples (Carnemolla et al., 2009). There is also an increase in levels of phosphorylated IRE1, GRP78 and XBP1 in striatal tissue of HD patients (Lee et al., 2012). Another study showed that toxic poly-glutamine expanded protein entraps ERAD proteins, leading to impairment of ERAD (Kalathur et al., 2015). Additional studies confirmed the weakened processing of ATF6 in both animal models and human HD patients (Fernandez-Fernandez et al., 2011). The proteinopathy of HD also targets autophagy by making it dysfunctional (Martin et al., 2015). The activation of JNK pathway in studies of cells overexpressing poly-glutamine accumulations further reinforces data showing UPR^ER^ driven neuronal death in HD (Kouroku et al., 2002).

## Remediation of Neurodegeneration by Targeting UPR^ER^

Being the underlying cause of aforementioned neurodegenerative diseases, the molecular signatures of UPR^ER^ are striking targets for therapeutic intervention. In mouse models of AD, chemical chaperones, 4-phenylbutyric acid (PBA) or tauroursodeoxycholic acid (TUDCA), have been shown to recuperate ER folding ability thereby rescuing neurons (Ricobaraza et al., 2012; Ramalho et al., 2013). Studies in human tau expressing stable cells (HEK293/tau) with overexpression of nucleotide exchange factor SIL1, a co-chaperone for GRP78, showed reduced tau hyperphosphorylation (Liu et al., 2016). However, a seemingly contrasting study showed that SIL1 might function in GRP78 independent manner for the amelioration of neuronal fitness in AD (Labisch et al., 2017). Dantrolene, licensed for treatment of spasticity, diminishes memory deficit by inhibiting PERK/eIF2α/CHOP in mouse AD models (Peng et al., 2012). Salubrinal (Sal), which selectively activates the levels of eIF2 α-P, causes elevation in GRP78 expression thereby protecting against Aβ neurotoxicity (Lee et al., 2010). In a similar way, Salubrinal also attenuates apoptosis that accentuates neuronal survival in PD mouse models (Colla et al., 2012; Mollereau et al., 2016). Out of the select set of mRNA translated after PERK-mediated phosphorylation of eIF2α, β-site APP cleaving enzyme-1 (BACE1) mRNA encodes for the key secretase that leads to the production of Aβ through the cleavage of amyloid precursor protein (APP; Kimura et al., 2016). Targeting the dephosphorylation of eIF2α-P by arctigenin, a bioactive product from *Arctium lappa*, leads to cessation of Aβ formation (Zhu et al., 2013). The restoration of translation and thereby prevention of neuronal loss was reported in mutant tau-expressing mice after challenging with PERK inhibitor, GSK2606414 (Radford et al., 2015). An attempt to target ISR, where eIF2α competitively inhibits eIF2B, an ISR inhibitor called ISRIB (affecting eIF2B) when administered in rodents, led to reversal of global translational halt that consequently enhanced long-term memory (Sidrauski et al., 2015). Emerging concepts suggesting different consequences of UPR^ER^ have highlighted advantageous role of XBP1 on memory. The administration of XBP1(S) through adeno-associated virus rescued long-term hippocampus memory in XBP1 knockout mice (Martínez et al., 2016). Quercetin, a flavonol, stimulates IRE1 endoribonuclease activity hence inhibiting tau hyperphosphorylation (Suganthy et al., 2016). Inhibition of autophagy by mammalian target of rapamycin (mTOR) complex (mTORC1) is challenged by AVN-211 (mTOR inhibitor) that concomitantly induces autophagic clearance of toxic aggregates in AD (Cai et al., 2015; Towers and Thorburn, 2016). Activation of XBP1 and ATF6 have also been proven to be protective in PD models (Egawa et al., 2011; Valdés et al., 2014; Mollereau et al., 2016). The suppression of PERK/eIF2α arm also prevented neurodegeneration in PD *Drosophila* mutants (Celardo et al., 2016). Several studies ascribe neuroprotective role of ATF6 (precisely, ATF6 α subtype) in PD mouse models (Hirsch et al., 2013; Voutilainen et al., 2015). One latest report proposes therapy for PD that activates Nrf2 through oral gavage of dimethyl fumarate (DMF) in mouse models (Lastres-Becker et al., 2016). Reports from studies in HD transgenic mice suggest that the selective silencing of XBP1 by small interfering RNA (siRNA) leads to mitigation of neural loss (Vidal et al., 2012). Additionally, XBP1-deficient HD transgenic mice showed augmented clearance of mHtt through autophagy (Vidal et al., 2012). Alleviation of ER stress by using chemical chaperone TUDCA in HD model showed reduction in neural loss and improved motor activity (Keene et al., 2002). The inhibition of eIF2 α-P/PERK rescued striatal neurons from huntingtin cytotoxicity (Leitman et al., 2014). The IRE1/JNK route of UPR^ER^ upregulates an actin binding protein, which is a negative regulator of autophagy, called ectodermal-neural cortex 1 (ENC1). ENC1 knockdown data illustrated relief in mHtt induced neuronal death (Lee et al., 2016). In recent development on transgenic *Drosophila*, treatment with autophagy-enhancing molecule, AUTEN-67, caused impediment in HD symptoms (Billes et al., 2016).

## Can We Interfere with Aging to Prevent Neurodegenerative Diseases by Targeting UPR^ER^?

We can look for the answer to this question by revisiting a remarkable study of the insulin/IGF-like signaling (IIS) pathway mutant of* C. elegans* that revealed lifespan extension was dependent on the active participation of IRE1 and XBP1, as was gaining resistance against ER stress. Additionally, XBP1 coordinated with IIS-activated forkhead box (FOXO) transcription factor DAF-16 to regulated important gene targets of longevity and ER homeostasis (Henis-Korenblit et al., 2010). Dietary restriction (DR) that promoted longevity and slowing of disease onset also showed stimulation of IRE1 in specific neurons of *C. elegans* (Chen et al., 2009). Reports from a forward genetic study of *C. elegans* again strengthen the stand of healthy ERAD and ensuing autophagy in life span extension and amelioration of proteinopathy maladies (Denzel et al., 2014). Thus, we can target neuronal damage that stems from aging.

## Conclusion

The ER plays an important role in the proper functioning and health of neuronal network. An aging system undergoing diminishing cellular performance enervates the ER, leading to decreased UPR^ER^, thereby failing to recuperate the imposed stress. Studies of model organisms have reinforced the importance of the activation of UPR^ER^ molecular markers in stimulating longevity. Age related dysfunction in UPR^ER^ weakens the ERAD pathway, thereby potentially promoting accumulation of misfolded protein cargo, which eventually becomes toxic intracellular inclusions. As witnessed by the studies highlighted in this review, the prominent aging driven neurodegenerative diseases share a common pathology of toxic misfolded protein accumulations. This provides an opportunity for therapeutic interventions to prevent the various molecular signatures of UPR^ER^ pathway that can stave off both aging and neuropathologies. Our focus on interfering with the temporal expression patterns of UPR^ER^ molecular markers can help us understand the unsolved issues of aging driven neurodegenerative diseases.

## Author Contributions

SR, JihK and RM conceived the idea; SR, ATJ, AA, JiwK, JihK and RM contributed to writing of the manuscript.

## Conflict of Interest Statement

The authors declare that the research was conducted in the absence of any commercial or financial relationships that could be construed as a potential conflict of interest.
